# The joint effects of forest habitat area and fragmentation on dung beetles

**DOI:** 10.1002/ece3.10429

**Published:** 2023-08-25

**Authors:** David Nyaga Mugo Mbora, Morris Nzioka Mutua

**Affiliations:** ^1^ Department of Biology, The Program in Environmental Science Whittier College Whittier California USA; ^2^ Tana River Primate National Reserve Hola Kenya; ^3^ Entomology Section, National Museums of Kenya Nairobi Kenya

**Keywords:** Africa, conservation, edge effects, habitat fragmentation, insect apocalypse

## Abstract

Habitat loss and habitat fragmentation usually occur together, at the same time and place. However, while there is a consensus that habitat loss is the preeminent threat to biodiversity, the effects of fragmentation are contentious. Some argue that habitat fragmentation is not bad for biodiversity, and even that it is good. Generally, the studies that find no harm or positive outcomes of fragmentation invariably assume that it is independent of habitat loss. However, dissociating the effects of habitat fragmentation from habitat loss is questionable because the two are essentially coupled. Accordingly, we evaluated how forest area and fragmentation (via edge effects) influenced dung beetles per se, and through their effects on the abundance of mammals, using structural equation modeling (SEM). Dung beetles are very sensitive to forest habitat loss and fragmentation and to changes in the abundance of mammals on which they depend for dung. Our study area was in the Tana River, Kenya, where forest fragments are depauperated of mammals except for two endemic species of monkeys. We mapped 12 forests, counted the resident monkeys, and sampled 113,955 beetles from 288 plots. Most of the 87 species of beetles found were small tunnellers. After implementing a fully latent Structural Regression SEM, the optimal model explained a significant 26% of the variance in abundance, and 89% of diversity. The main drivers of beetle abundance were positive, direct, effects of forest area and number of monkeys, and negative edge effects. The main drivers of diversity were the direct effects of the beetle abundance, indirect effects of forest area and abundance of mammals, and indirect negative edge effects. Thus, forest area, fragmentation (via edge effects), and the number of monkeys jointly influenced the abundance and diversity of the beetles directly and indirectly.

## Graphical abstract

We evaluated how forest area and fragmentation (via edge effects) influenced dung beetles per se, and through their effects on the abundance of mammals, using structural equation modeling (SEM). The optimal model explained a significant 26% of the variance in abundance, driven by the positive, direct, effects of forest area and number of monkeys, and negative edge effects. Further, the model explained 89% of the variance in diversity, driven by the direct effects of the beetle abundance, indirect effects of forest area and abundance of mammals, and indirect negative edge effects.

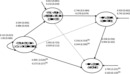

## 1 INTRODUCTION

Habitat loss is the main cause of the depletion of biodiversity (Ceballos et al., 2015; Dirzo et al., 2014), but the effects of the ensuing fragmentation are contentious (Miller‐Rushing et al., 2019). Several studies question the idea that habitat fragmentation is a bad thing for biodiversity (Fahrig, 2017; Fahrig et al., 2019), but other studies show that habitat fragmentation is a major cause of species loss and eventual extinctions (Haddad et al., 2015, 2017). In general, the studies that find no harm and/or positive effects of habitat fragmentation invariably assume that it is distinct from habitat loss. However, dissociating habitat fragmentation from habitat loss is questionable because the two processes are inherently linked (Didham et al., 2012; Fletcher et al., 2018; Püttker et al., 2020). It can be informative, therefore, to test what the joint, coincident, effects of habitat loss and habitat fragmentation are on the abundance and diversity of species.

The effects of habitat loss and fragmentation on species can be direct and indirect, at the same time. Directly, habitat loss drives many species to their extinction threshold (Fahrig, 2002), and the most vulnerable are those with poor abilities for dispersal and/or specialist resource needs (Henle et al., 2004). Indirectly, the breaking apart of the habitat and increased isolation of remnant patches (i.e., fragmentation) increase the edge effects and impact the resident species in many ways (Fahrig, 2003; Haddad et al., 2015; Murcia, 1995). Another major indirect effect is that fragmentation can disrupt the ways in which species interact (Hagen et al., 2012). Such disruptions are especially problematic for species with close associations with others due to knock‐on effects caused by the impacted interactants (Abrego et al., 2016; Fortuna & Bascompte, 2006). Thus, in any landscape, there are multiple ways by which habitat loss and fragmentation acting jointly could influence the abundance and diversity of species.

Three ecological concepts can offer a framework within which to test for the direct and indirect effects of habitat loss and fragmentation. They are the: (1) species‐area relationship (Arrhenius, 1921; Gleason, 1922), (2) individuals‐area relationship (Bender et al., 1998; Connor et al., 2000), and (3) the edge response model (Ries et al., 2017; Ries & Sisk, 2004).

The species‐area relationship is the general ecological pattern in which the total number of species found in a place increases with the area (Arrhenius, 1921; Gleason, 1922). The pattern exists because the larger an area is, then the more diverse the habitats it can contain and the greater the variety of niches that it can offer. Analogously, the individuals‐area relationship is the widespread occurrence of a positive association between animal population density and area (Connor et al., 2000; Gaston & Matter, 2002). The individuals‐area relationship originated from empirical observations in which it was found that habitat patches with large amounts of resources (e.g., monocultures, patches of high plant density, or larger patches) contained higher densities of insects (Root, 1973). Over the years, empirical studies (Connor et al., 2000; Gaston & Matter, 2002) have shown that the individuals‐area relationship can be due to one of three main mechanisms acting alone or in concert: (1) the resource concentration hypothesis (Root, 1973), (2) the movement hypotheses (Bach, 1988), and (3) the enemies hypothesis (Risch, 1981). For its part, the edge response model presents the two main mechanisms by which edge effects can influence the abundance and diversity of species (Ries et al., 2017; Ries & Sisk, 2004). In short, adjoining fragments may contain different resources. If fragments are quite small, the adjacency puts such resources within reach. Moreover, a variety of resources may accumulate along fragment edges, which if coupled with favorable conditions would increase the number of individuals, and species, that could thrive there.

Dung beetles in forests provide an appropriate study system in which to test for the joint, direct, and indirect effects, of habitat loss and fragmentation. Increasingly nowadays, dung beetles have become a choice taxon with which to study the ecology of habitat changes (Audino et al., 2014; Filgueiras et al., 2015; Noriega et al., 2021). As a group, these beetles are sensitive to forest modifications and fragmentation (Davis et al., 2001; Nichols et al., 2007), and changes in the abundance of mammals (Correa‐Cuadros et al., 2022; Raine & Slade, 2019). Thus, the widespread destruction of forest habitat is a serious threat to the beetles in and of itself (Foley et al., 2005; López‐Bedoya et al., 2022). That the destruction also wipes out the resident mammals multiplies the threat many times (Culot et al., 2013; Raine & Slade, 2019) because the beetles depend on the mammal dung as a main resource for food and nesting (Halffter & Edmonds, 1982; Hanski & Cambefort, 1991). Accordingly, there are many studies that have evaluated the effects of forest destruction on the dung beetles, but nearly all such studies tested the effects of forest loss as a separate thing from the effects of forest fragmentation (Fuzessy et al., 2021; Nichols et al., 2007). Therefore, it can be informative to test what the joint, direct, and indirect, effects of forest loss and fragmentation are on the dung beetles.

The Tana River forest in Kenya is one place where a growing population of people has cut and fragmented much of the habitat (Mbora & McPeek, 2009; Mbora & Meikle, 2004a). As a result, the forest is now just a fraction of what it once was, in a matrix of small‐scale farms and settlements, savannah shrubs, and woodland. The forests, however, are the only habitat of two species of endemic monkeys: the Tana River red colobus (*Procolobus rufomitratus*, Peters, 1879) and the Tana mangabey (*Cercocebus galeritus*, Peters, 1879). So we chose this study system, which we know well, to test for the joint direct, and indirect effects of habitat loss and fragmentation on the dung beetles. We aimed to answer this question: In what ways do forest area, abundance of mammals, and edge effects, acting jointly, influence the abundance and diversity of the dung beetles?

**FIGURE 1:**
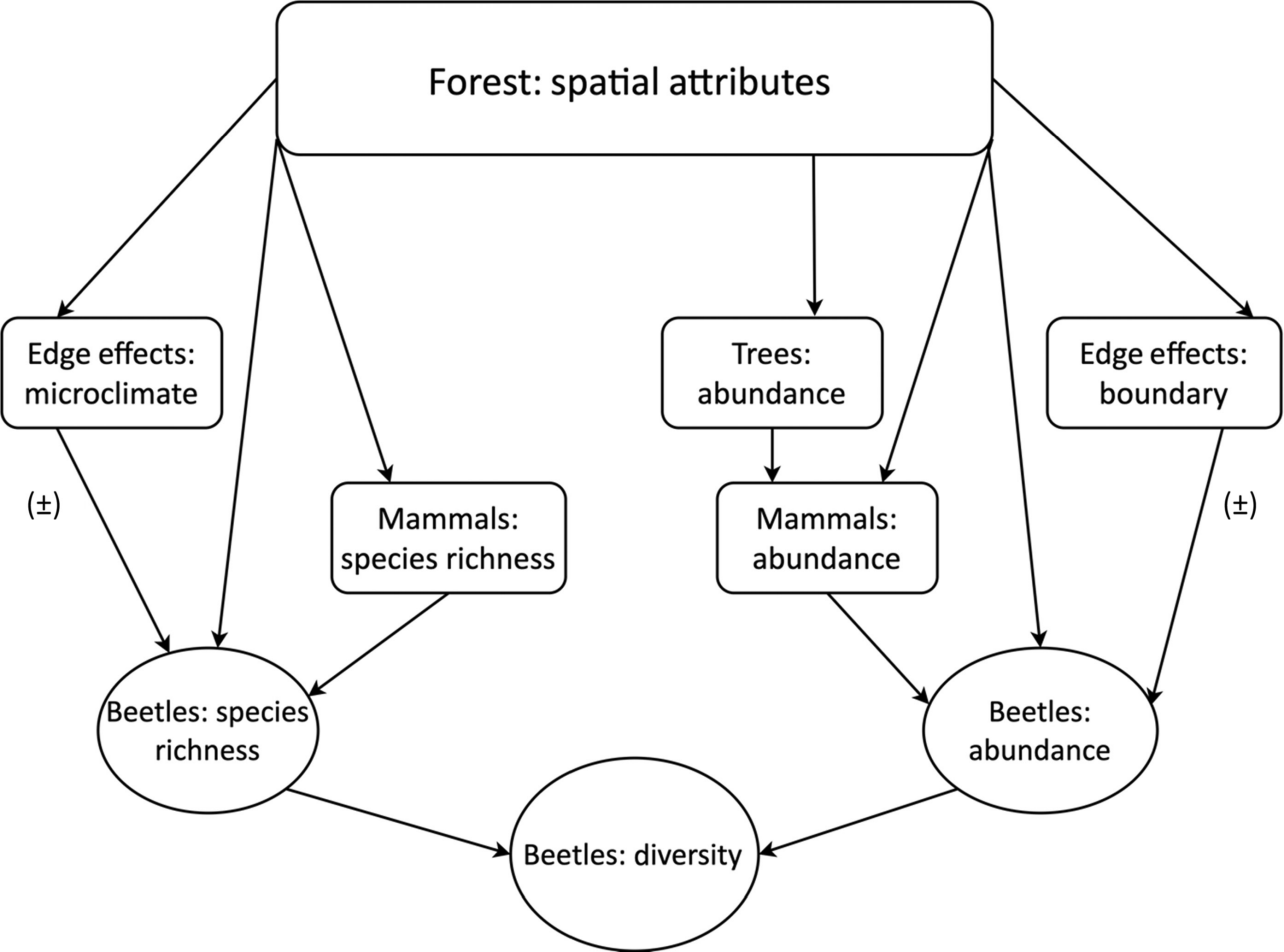
Conceptual meta‐model of postulates of the ways in which attributes of the forest habitat could influence the abundance and species richness, thus diversity of the dung beetles in forest fragments. Fragment areas could influence the abundance and species richness of the beetles directly. Simultaneously, the dimensional features of the fragments could influence the abundance and species richness of the beetles indirectly, through their effects on the resident mammals and edge effects. Lines with a single arrowhead represent hypothesized positive directional causal effects, except where indicated as unforeseeable (±), and could be positive or negative (details in the text).

There are several direct and indirect ways by which the forest area, the abundance of mammals, and the edge effects could influence the abundance and diversity of the dung beetles in our study system, and elsewhere (Figure 1). Directly, the amount of forest could influence the abundance and species richness of the beetles, through the species and the individual area relationships. Therefore, we predicted positive correlations between the forest area and both the abundance and species richness of the beetles (Fuzessy et al., 2021; von Hoermann et al., 2020). Further, the direct effects due to the individuals‐area relationship would apply to the forest trees and the monkeys which would in turn influence the beetles (Figure 1). In these forests, the area and abundance of the trees are directly correlated (Wieczkowski, 2004), and because the monkeys depend on the trees for food, then the number of monkeys is itself correlated with the abundance of the trees (Mbora & McPeek, 2009; Mbora & Meikle, 2004a). Therefore, we expected positive associations between the forest area, the abundances of the trees, and the monkeys, and thus in turn the monkeys would influence positively the abundance and diversity of the beetles. In a similar fashion, the species‐area relationship would apply to the mammals, which would in turn influence the species richness of the beetles (Figure 1).

Additional impacts on the beetles would come from edge effects (Figure 1), in accordance with the edge response model. First, the abundance and variety of mammals at the forest edge could be higher than in the interior (Brodie et al., 2015). This would most likely occur, for instance, if the savanna wildlife and/or regular livestock utilizes the habitat at, or near, the forest edge. Indeed, people could be a big factor also as they routinely use forest edges for toileting (personal observation, by DNMM). In these cases, the higher amounts, and varieties of dung at the edge could promote the abundance and species richness of the beetles. Conversely, increases in edge effects could reduce the habitat quality for the forest‐dependent monkeys, diminish dung resources, and, therefore, the abundance and species richness of the beetles. Second, forest edges are usually drier and hotter than the forest interiors are (Chen et al., 1999). As such, conditions at the edge could then favor the species of beetles associated with the more open matrix, and not so much the beetles associated with the closed forest. As a result, the assemblage of beetles at the edge could be a mix of beetles from each of these habitat types. In sum, the edge effects on dung resources and the microclimate mean that they would influence the abundance and species richness of the beetles somehow. However, the direction and magnitude of these effects are complex, and not easily discerned a priori (Figure 1).

To test our postulate rigorously, and answer the research question, we used Structural Equation Modeling, SEM for short (Grace, 2006; Kline, 2016). SEM is the proper tool for this analysis because it allows us to evaluate the direct and indirect effects concurrently (Figure 1). What's more, the testing of our predictions requires the use of covariates that cannot be observed and/or measured directly, but which with SEM can be analyzed as latent factors (Grace, 2006; Kline, 2016). This could then highlight processes that are important but are otherwise unremarked and point to areas in need of further studies, and perchance conservation interventions.

## 2 MATERIALS AND METHODS

### 2.1 Study area

**FIGURE 2:**
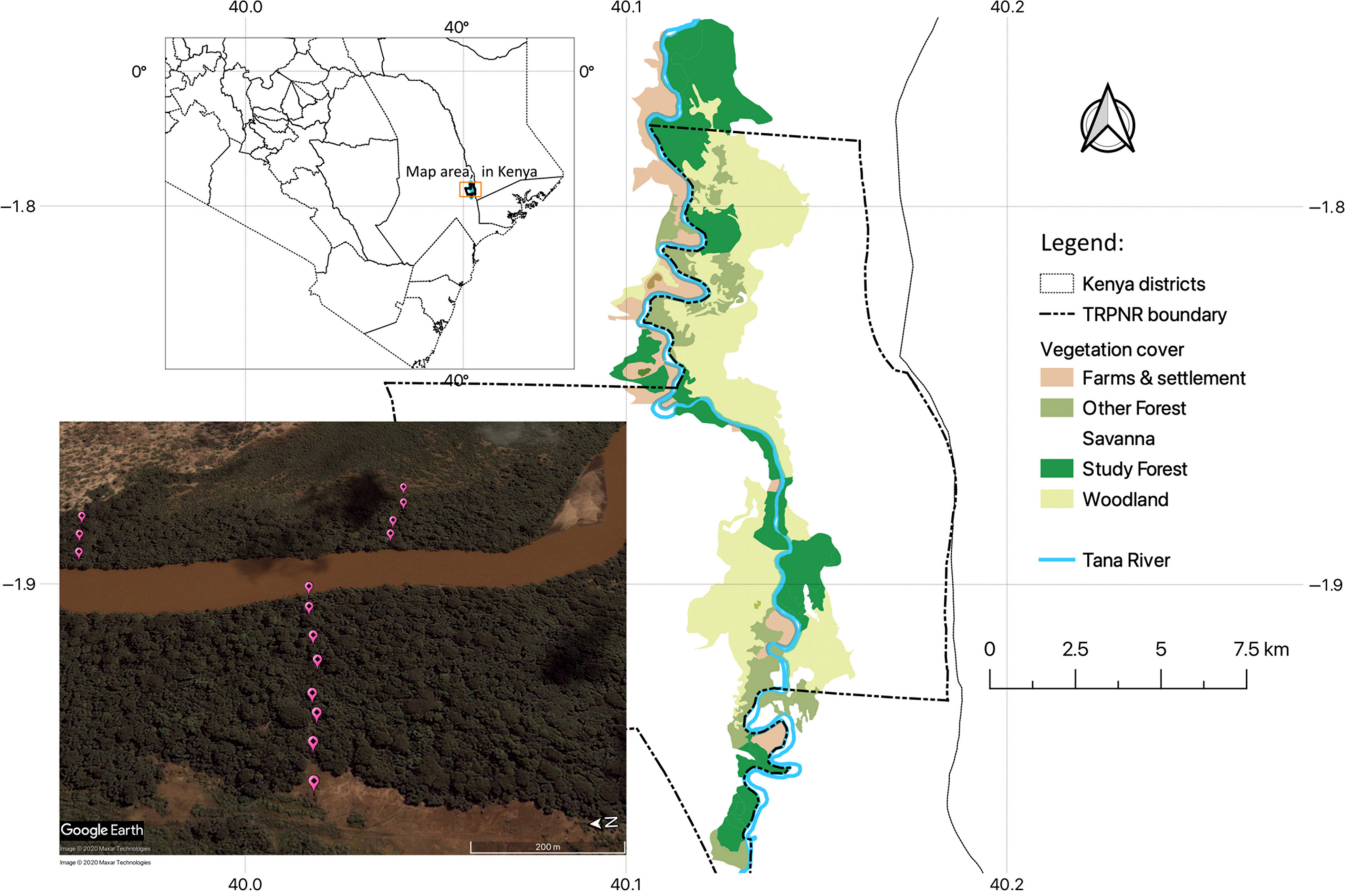
Map of the study area in Kenya, showing the distribution of the forests along the Tana River, (TRPNR stands for Tana River Primate National Reserve). Inset photograph (lower left) is a bird's‐eye view of the orientation of three illustrative transects in two forests on opposite banks of the River. Pushpins indicate the sampling stations.

This study was in a tropical forest remnant along the Tana River in Kenya (Figure 2), comprising fragments of various sizes, in a matrix of small‐scale farms and settlements, savannah shrubs, and woodland (Mbora & McPeek, 2009). Groundwater and occasional flooding maintain the forest, restricting it to a lateral extent of about a kilometer on either side of the river (Figure A1a). The forest supports a high diversity of rare plant and animal species (Andrews et al., 1975), and it is the only known habitat for the endemic Tana River red colobus and mangabey. The Tana River red colobus is a folivore, but it also feeds on a variety of other plant matter such as fruits and flowers (Marsh, 1981). As a colobine, the red colobus possesses a ruminant‐like stomach, and a digestive system, akin to herbivorous grazing or browsing mammals (Chivers, 1980; Davies & Oates, 1994). The mangabey for its part is a frugivore that feeds mostly on fruits and seeds, selecting the most abundant plant materials (Homewood, 1978). The typical diet of the mangabey comprises 44% fruit, 32% seeds, and the rest (24%) an assortment of stems, leaves, and miscellaneous items.

In addition to local people who also use the forests in various ways, there are three other species of primates: the yellow baboon (*Papio cynocephalus*, Linnaeus, 1766), Sykes' monkey (*Cercopithecus albogularis*, Wolf 1822) and the vervet (*Chlorocebus aethiops*, Linnaeus, 1766). However, these other species are not forest‐dependent and typically use the matrix habitat. Otherwise, the forests are depauperate of large mammals (Andrews et al., 1975).

### 2.2 Sampling of the beetles

We focused on 12 forest fragments within the defunct Tana River Primate National Reserve (TRPNR; Figure 2; 1.9346° S, 40.1349° E). Seven fragments were located on the west bank of the river and the other five on the east. We collected dung beetles from sampling stations located along permanent transects, in each forest, using standardized pitfall trapping (Larsen & Forsyth, 2005). Fieldwork was completed over June, and July in 2009, 2010, 2011, and 2014, when we sampled transects only once each time.

The location of the transects in the fragments was systematic, aligned perpendicular to the river, and ran through to the edge adjacent to the savanna (Figures A1a and A1b). This alignment captured the main gradient in forest community composition;‐ a decreasing basal area of forest trees with increasing distance from the river (Mbora & Meikle, 2004a). We established sampling stations every 50 m along each transect (Larsen & Forsyth, 2005), with one station located 50 m into the savanna (Figure 2, Figure A1b). Because of this systematic alignment of the transects, the total number of sampling stations (Table A1) was proportional to area, but it was also dependent on the inherent variation in the vegetation of the forest.

The sampling station was a quincunx of pitfall traps. Each trap was a screw‐cap plastic specimen jar of 236 mL volume, containing 100 mL of water laced with an odorless detergent to break the surface tension. Traps were buried flush with the soil surface and baited with fresh cow dung (≈5–10 g) wrapped in cheesecloth. We used cow dung as bait because it is exceedingly difficult to get any amount of dung from the endemic primates, which we knew from earlier studies (e.g., Mbora & McPeek, 2009, 2015). Fortunately, dung beetles are generalist consumers of mammalian dung.

The whole setup was shaded with a plastic plate, held at 20 cm above with yard staples, and left in the field for 24 h. Upon recovery, we separated the dung beetles from everything else and preserved them in 70% ethanol. Then, we identified the beetles to the level of species or morphospecies using the dung beetles reference collection at the National Museums of Kenya (Invertebrates Zoology section), supplemented with other taxonomic keys as needed, primarily (Scholtz et al., 2008). We recorded the number of individuals in each trap and determined the mass (in milligrams) of the beetles in the following way. For each species, we made two‐point measurements from head to pygidium of 15–10 individuals with a digital microscope (Keyence VHX, America). We then calculated an average body length, converted the average length into body mass using the power function (Lobo, 1993), and then multiplied the number of individuals of each species in each pitfall to give the total mass. We used the power function, rather than the linear logarithmic transformation because our samples included notable variations in size and shape (Lobo, 1993).

In dung beetles, behavioral (nesting) strategies and body size influence vulnerability to forest loss and fragmentation (Nichols et al., 2013). Accordingly, we applied the three standard behavioral categories for each species: dwellers nest within the pile, tunnellers dig burrows underneath, and rollers fashion a dung ball which they push and bury a distance away (Doube, 1990; Hanski & Cambefort, 1991; Scholtz et al., 2008). As for body size, we classified the species in each pitfall into three classes of length in mm (small: 2 ≤ 6; medium: >6 ≤ 10; large >10), heuristically as follows. First, we generated a density plot of the mass (mg) of all beetles in the data set, which revealed three distinct peaks (Figure A2a). Then, we used the range of values of biomass (minimum—maximum) of each peak to designate the three size classes (Figure A2b), by back‐transforming the mass into length (Lobo, 1993).

### 2.3 Data syntheses and analyses

#### 2.3.1 Dung beetles: Abundance and diversity

We optimized the data set by pooling the five traps of a sampling station into a “sampling occasion,” constituting one visit to one sampling station during a sampling season (i.e., June and July in 2009 vs. 2010 vs. 2011 vs. 2014). This resulted in 288 “sampling occasions,” to which we refer henceforth as “plots,” comprising the number and mass (mg) of the beetles (Table A1). Nine transects from the east of the river contained 171 plots and 12 transects from the west contained 117 plots. Then using the number of individuals as a measure of abundance, we calculated the diversity and assessed the sample completeness as coverage per plot with iNEXT version 3.0.0 in R (Hsieh et al., 2016). We computed diversity in Hill numbers (i.e., q = 0, 1, 2) as non‐asymptotic, and as the asymptotic or true, diversity of each plot. In addition, we estimated the point diversity at a 95% coverage in the sense of a confidence interval, that is, 95% of the estimates contained the true diversity of the plot.

#### 2.3.2 Habitat attributes: Forests, monkeys, and trees

We previously mapped the 12 study forests and determined their metrics for earlier projects (Mbora & McPeek, 2009, 2015; Mbora & Meikle, 2004b). For each forest, we recorded the area, perimeter, area‐to‐perimeter ratio, and centroid distance to the matrix (Table A1). Then, we surmised that the number of endemic monkeys was the best measure of the abundance of mammals in the forests. Therefore, we adopted the data of the monkeys from our earlier projects (Mbora & McPeek, 2009, 2015; Mbora & Meikle, 2004b). These data constituted the number of individuals, and social groups, per forest for each species of monkey (Table A1). This adoption was justified because these forests are depauperate of large mammals, and illegal hunting eliminated elephants from the contiguous savanna habitat by the 1980s (Allaway, 1979; Andrews et al., 1975). Advantageously the colobus and mangabey are forest‐dependent and we had good quality data on their abundance from the earlier projects (Mbora & McPeek, 2009, 2015; Mbora & Meikle, 2004b) and our field records (David N. M. Mbora, unpublished data).

Because the endemic monkeys are forest dependent, their abundance is correlated positively with the abundance of living trees, and negatively with an abundance of trees cut or damaged by people for consumptive use (Mbora & Meikle, 2004b; Wieczkowski, 2004). Therefore, we also measured the abundance of living trees, and trees cut or damaged by people, along transects as we did previously (Mbora & McPeek, 2009; Mbora & Meikle, 2004b). We then summarized the abundance of trees as basal area (cm^2^) of living trees, and of the trees that were cut, or damaged, per forest (Table A1).

#### 2.3.3 Specification of SEM factors

**FIGURE 3:**
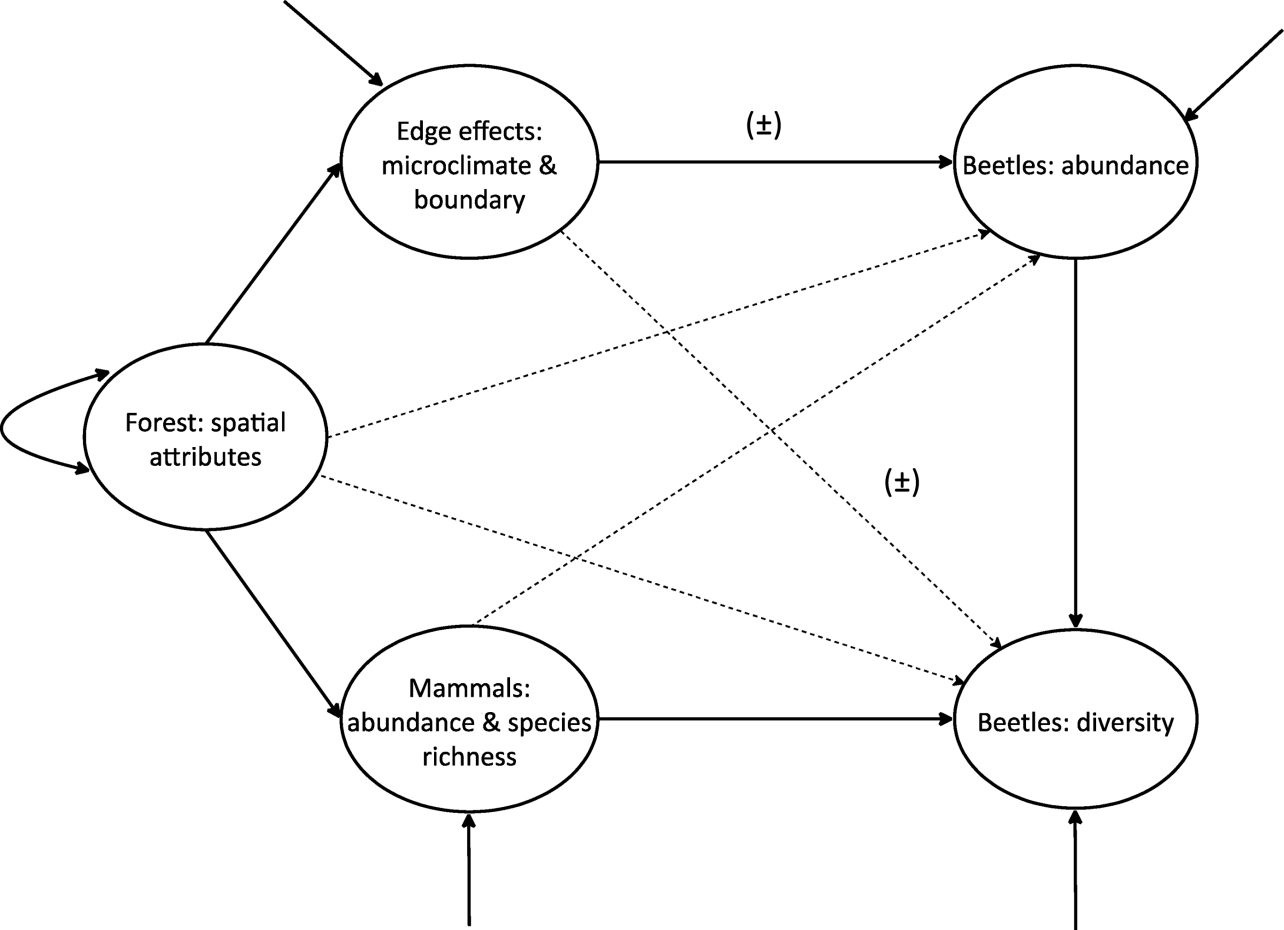
Structural component (in compact symbolism) of the starting structural regression model of the effects of habitat variables on the beetle abundance and diversity of the dung beetles. The single inward‐pointing arrow represents exogenous variances and endogenous disturbances. Lines with a single arrowhead represent hypothesized directional positive causal effects, except where indicated as (±), which could be positive or negative (see text for details). Solid lines represent the postulated main causal effects.

Following our postulates (Figure 1), we specified a starting latent Structural Regression (SR) model with three factors, which would influence the abundance and diversity of the beetles (Figure 3). The factor “Forest: spatial attributes,” encapsulated the ways in which the area and other dimensional features of the forests could influence the beetles. In turn, the factor “Mammals: abundance & species richness,” encapsulated the effects of the mammals, including how the forest trees influenced the monkeys. The factor “Edge effects: microclimate & boundary,” encapsulated the ways in which edge effects influenced conditions and resources in the forests and their impact on the dung beetles.

Two latent factors encapsulated the abundance and diversity of the dung beetles. The factor “Beetles: abundance,” represented the number of individuals or the mass of all species of beetles. And “Beetles: diversity,” encapsulated a composite of the species richness and the abundance of the beetles. So in this specification, we did not conceptualize species richness and diversity as separate entities because richness and standard measures of diversity (e.g., Shannon and Simpson indices) are special cases of one general equation, denominated in units of species through Hill numbers (Roswell et al., 2021). Fundamentally however, diversity is a composite of the number of species in a community and their abundances, and the two factors are correlated (Figure 1).

#### 2.3.4 Selection of SEM factor indicators

SEM analyses require that data must have normal distributions (Kline, 2016). However, upon assessing the normality of all variables using histograms and q‐q plots (Mishra et al., 2019) we found that only species richness (Hill.q0) was normally distributed. Therefore, we transformed all other variables to approximate normality.

Multiple variables in the data set could serve as the reflective indicators of each factor in the SEM (Figure 3). Therefore to select an optimal set of indicators for each factor, we computed a correlogram for habitat variables (Figure A3a) and another for the beetles (Figure A3b). Then, we followed recommendations of best practices in SEM (Kline, 2016) to select the optimal indicators of each factor. Forest area emerged as the optimal factor for “Forest: spatial attributes,” that is, a single indicator factor. Number of colobus and mangabey combined, and number of mangabeys were optimal for “Mammals: abundance & species richness.” Perimeter of the forest, distance of a plot to the matrix, and the centroid distance of the fragment to the matrix were optimal for “Edge effects: microclimate & boundary.” Mass of the beetles separated into tunneling, medium and large for “Beetles: abundance.” And species richness (Hill.q0), Shannon diversity (Hill.q1), and asymptotic species richness (Hill.q0) for ‘Beetles: diversity.’

#### 2.3.5 SEM fitting and estimation

In keeping with the best practices of SEM, we implemented a two‐step modeling (Kline, 2016) in lavaan version 0.6‐6 using R (Rosseel et al., 2017). We started by specifying the structural regression (SR) model as a Confirmatory Factor Analyses model to ensure that the measurement part was consistent with the data. Then, we fit and estimated the fully latent SR model using the default maximum likelihood estimator. In model fitting, classical SEM theory assumes that all data are independently and identically distributed (iid). However, our sampling framework violated this iid assumption because the plots were nested within forests, and the forests themselves were nested east or west of the river (Figure 2, Figure A1b). So we accounted for this nestedness by coding the initial fit object from lavaan with an object specifying the sampling framework in lavaan.survey (Oberski, 2014) to estimate and fit the final models.

We evaluated the acceptability of the final models using a global fit and three associated fit indices. A satisfactory model was indicated by a non‐significant χ^2^‐statistic (*p* > .05), but we also applied the alternative *F*‐reference distribution at the step where the sampling framework is specified in lavaan.survey (Oberski, 2014). In addition, a satisfactory model needed to attain the following thresholds of fit indices: comparative fit index, CFI > 0.90; root mean square error of approximation, RMSEA < 0.05 (for lower 90% confidence intervals); a standardized root mean square residual, SRMR < 0.10. Lastly, we estimated the power of the final SR models as: the tests of close‐fit‐hypothesis (H_0_: ε_0_ ≤ 0.05, H_1_: ε_1_ = 0.08), and not‐close‐fit hypothesis (H_0_: ε_0_ ≥ 0.05, H_1_: ε_1_ = 0.01) (Preacher & Coffman, 2006).

We assessed the significance of the individual factors in the final models using the standardized path coefficients (β) and their *p‐*values. Then, we used the path coefficients to calculate the direct, indirect, and total effects of the latent factors on the endogenous variables (Kline, 2016). Lastly, we used the coefficients of determination (*R* ^2^) to assess how much of the variance of each endogenous variable was because of the respective exogenous variables.

## 3 RESULTS

**FIGURE 4:**
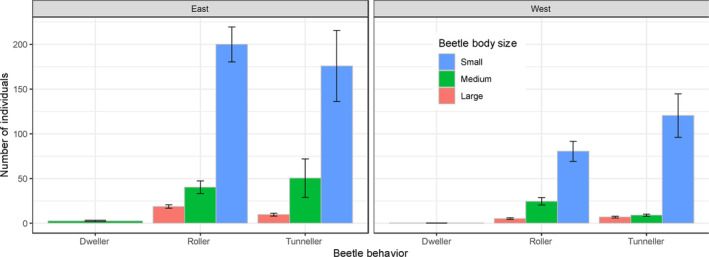
The number of dung beetles (95% CI) found per plot east and west of the Tana River, classified by behavior (nesting strategies) and body size. Most of the beetles were of small body size.

**FIGURE A5:**
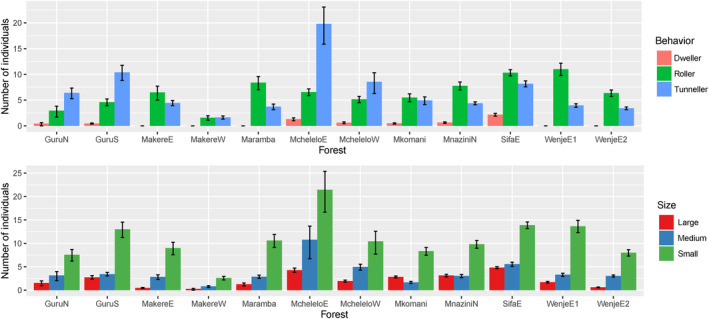
Mean number of dung beetles per plot in each forest, summarized by behavior (top panel), and body size (bottom panel).

**FIGURE A6:**
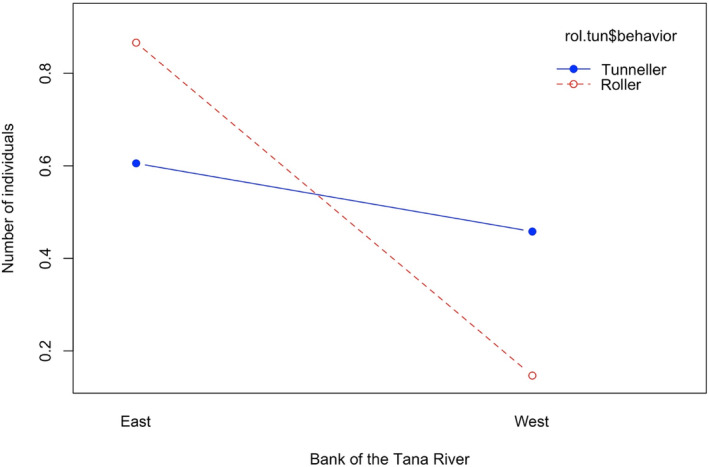
The mean abundance of beetles per pitfall east and west of the river, categorized by nesting strategy. On the Y axis, the number of individuals is expressed as log_10_.

**FIGURE A7:**
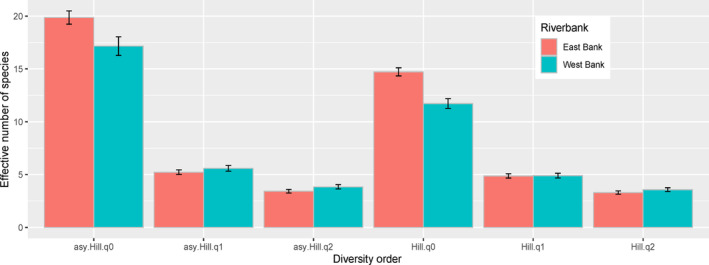
Dung beetle diversities (95% CI), east and west of the Tana River.

We identified 87 species from 113,955 dung beetles in 288 plots sampled from 21 transects in the 12 forest fragments (Tables A1 and A2). Sampling was complete in all forests (Figures A4a and A4b). Most of the species were of small or medium body size (80 of 87, 92%), of which most were tunnellers (68 of 87, 78%) but very few dwellers (Figure 4, Figure A5). Overall, there was no difference in the total number of beetles east and west of the river, rollers were more abundant on the east bank and tunnellers on the west (two‐way ANOVA, *F* (1,1724) = 5.71, *p* = .02; Figure A6). The diversity (Hill.q1 or Hill.q2) of all beetles was the same east and west of the river (Figure A7).

The starting model with five factors (Figure 3) converged normally and did not significantly deviate from the data (χ^2^ = 67.25, df = 56, *p* = .14; Table A3). However, this solution was inadmissible due to Heywood cases (Kline, 2016), produced by a poor discriminant validity between the factors “Forest: spatial attributes” and “Mammals: abundance and species richness” (covariance = 0.90, SE = 0.014; Tables A4 and A5). Therefore, we trimmed the model to four factors and combined the spatial and resource attributes into one factor “Forest and mammal abundance,” with indicators such as forest area, number of colobus and mangabeys combined, and number of mangabeys. This re‐specification was conceptually sound because the dimensional attributes of the forests and the resident mammals must influence the abundance and diversity of the beetles simultaneously (Figure A3a). The other factors in the SEM, and their indicators, remained the same as specified above.

**FIGURE A8:**
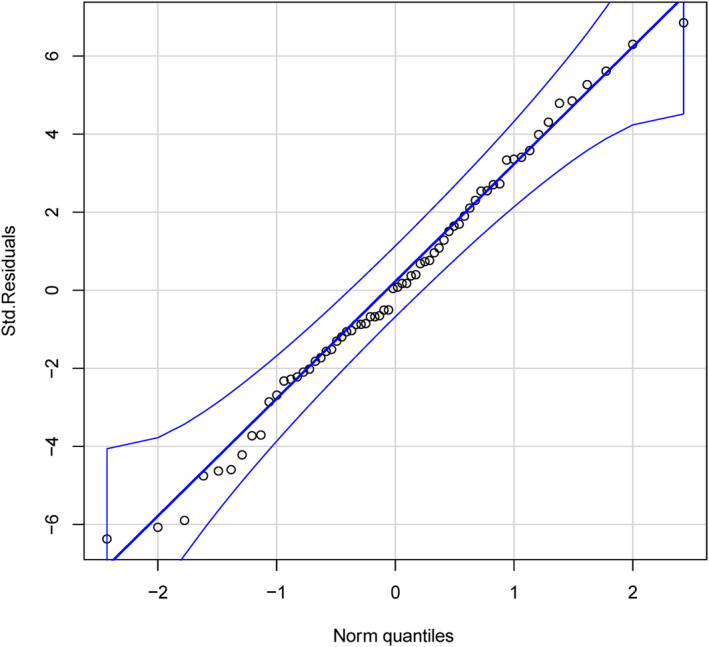
Quantile plot of standardized residuals of the four‐factor structural regression model of the determinants of dung beetle abundance and diversity.

The respecified model converged normally to an admissible solution, which did not significantly deviate from the data (χ^2^ = 72.33, df = 48, *p* = .44; Table 1). This model explained most of the variance (>65%) in nine of the 12 indicators of the four factors, demonstrating good convergent validity (Table 2), and which was supported by the pattern of a normal distribution of correlation residuals (Table A6; Figure A8). Furthermore, four of the six standardized factor correlations *R* ^2^ were well below 0.50 indicating reasonable discriminant validity (Table 3).

**TABLE 1:** Global fit statistics of a two‐step testing of the four‐factor structural regression model of the determinants of dung beetle abundance and diversity.

Model (*N* = 288)	χ^2^	df	*p*	χ^2^_*D*_	df_D_	*p*	RMSEA (90% CI)	CFI	SRMR
Measurement model, abundance & diversity:									
One factor—CFA	1269.95	54	.00				0.28 (0.27–0.29)	0.54	0.21
Four factors—CFA	380.84	48	.00	889.11	6	.00	0.16 (0.14–0.17)	0.87	0.08
Four factors—CFA (ids = ~east. west)	72.33	48	.01 (.44*)	308.51	0	.00	0.04 (0.03–0.05)	0.97	0.07
Structural regression model, abundance & diversity									
Three paths, unnested (ML)	380.84	48	.00				0.16 (0.14–0.17)	0.87	0.074
Nested (ids = ~forest)	105.52	48	.00 (.11*)				0.07 (0.06–0.07)	0.81	0.074
Nested (ids = ~east. west)^a^	72.33	48	.01 (.44*)				0.04 (0.03–0.05)	0.97	0.074

* *p*‐Value from the *F*‐reference distribution; *ids* specifies the survey design.

^a^ Indicates retained model.

**TABLE 2:** Robust maximum likelihood estimates of pattern coefficients and residuals of a four‐factor measurement model of the determinants of dung beetle abundance and diversity.

	Pattern coefficients				Error variances			
	Unstandardized		Standardized		Unstandardized		Standardized	
Factors and Indicators	Estimate	SE	Estimate	SE	Estimate	SE	Estimate	SE
Dung beetle abundance (mass, mg.)								
Large body size	1.00	—	0.69	0.04	1.38	0.23	0.53	0.06
Tunneling behavior	0.59	0.00	0.84	0.02	0.18	0.04	0.30	0.04
Medium body size	0.47	0.00	0.813	0.03	0.14	0.04	0.34	0.04
B Dung beetle diversity								
Hill.q0	1.00	—	0.98	0.03	1.14	1.86	0.04	0.07
Asymptotic Hill.q0	0.04	0.01	0.81	0.07	0.02	0.00	0.35	0.12
Hill.q1	0.02	0.00	0.40	0.05	0.05	0.00	0.84	0.04
C Forest area and mammal abundance								
Forest area (m^2^)	1.000	—	0.94	0.00	0.02	0.00	0.11	0.00
Colobus & mangabey, individuals	0.92	0.06	0.94	0.02	0.01	0.00	0.11	0.04
Mangabeys, individuals	0.86	0.23	0.82	0.07	0.04	0.04	0.32	0.12
D Edge effects: microclimate & boundary								
Absolute distance to matrix	1.000	—	0.49	0.01	0.17	0.02	0.76	0.01
Centroid distance to matrix	18.45	0.24	0.60	0.07	33.30	12.96	0.64	0.08
Forest perimeter	1.39	0.10	0.95	0.09	0.01	0.02	0.09	0.17

**TABLE 3:** Robust maximum likelihood estimates of factor variances and covariances for a four‐factor measurement model of the determinants of dung beetle abundance and diversity.

Parameter	Unstandardized		Standardized	
	Estimate	Standard error	Estimate	Standard error
Dung beetle abundance (DBA)	1.25	0.07	1.00	0.00
Dung beetle diversity (DBD)	26.20	0.89	1.00	0.00
Forest area and mammal abundance (FAMA)	0.12	0.02	1.00	0.00
Edge effects: microclimate & boundary (EE)	0.06	0.00	1.00	0.00
DBA ~~ DBD	5.37	0.37	0.94	0.05
DBA ~~ FAMA	0.17	0.00	0.43	0.04
DBA ~~ EE	0.07	0.01	0.25	0.03
DBD ~~ FAMA	0.66	0.02	0.37	0.03
DBD ~~ EE	0.31	0.05	0.26	0.05
FAMA ~~ EE	0.07	0.01	0.89	0.01

Given *N* = 288, df = 48, and α = 0.05, the power for the test of the close‐fit‐hypothesis (H_0_: ε_0_ ≤ 0.05, H_1_: ε_1_ = 0.08), was 0.91, and that for the not‐close‐fit hypothesis, (H_0_: ε_0_ ≥ 0.05, H_1_: ε_1_ = 0.01), was 0.83. Therefore, the probability of rejecting a false model, or detecting a correct one was good.

**FIGURE 5:**
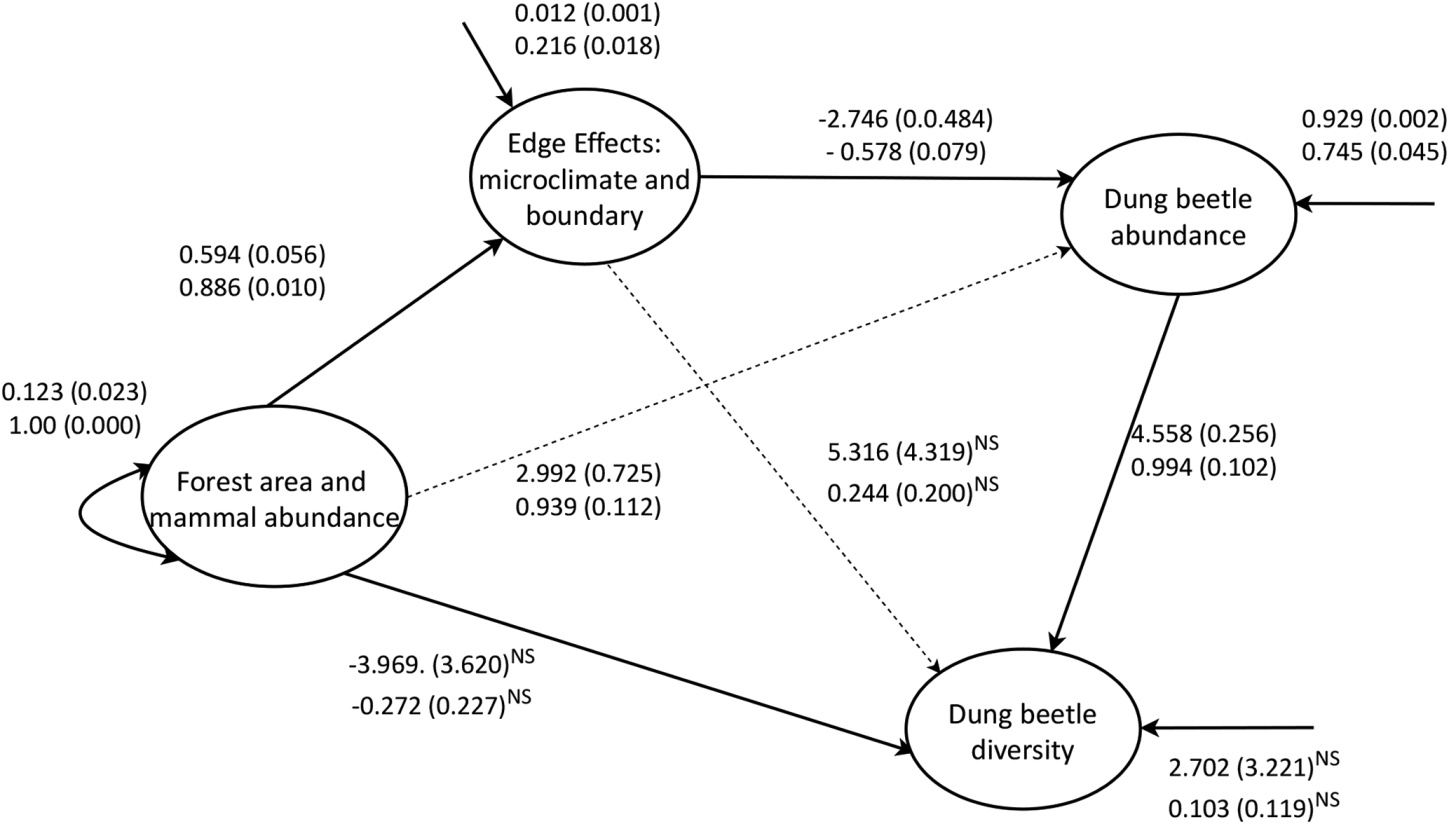
The structural component of the structural regression model of the effects of habitat attributes on dung beetle abundance and diversity, with disturbances presented in compact symbolism. The top row is unstandardized estimates (Standard error), and the bottom row is standardized estimates (Standard error), from a completely standardized solution. All estimates are statistically significant (0.05 level) except where indicated “NS” (not significant).

The standardized effect decompositions (Table 4; Figure 5) revealed that the direct effects of forest area and mammal abundance were positive on edge effects (ß = 0.89, SE = 0.01, *p* = .00; *R* ^2^ = .75) and the beetle abundance (ß = 0.94, SE = 0.11, *p* = .00). However, the direct relationship between edge effects and the beetle abundance was negative (ß = −0.58, SE = 0.08, *p* = .00), as were the indirect effects of forest area and mammal abundance (ß = −0.51, SE = 0.08, *p* = .00). Nevertheless, the total effects of forest area and mammal abundance were positive and statistically significant (ß = 0.43, SE = 0.04, *p* = .00). The model explained one quarter of the variance in the abundance of the beetles (*R* ^2^ = .26).

**TABLE 4:** Standardized effect decompositions (standard errors) of the structural component of the structural regression model of the effects of forest and mammal abundance, and edge effects, on dung beetle abundance and diversity (see Figure 5).

Endogenous factor	Effect	Forest area and mammal abundance	Edge effects	Dung beetle abundance
Edge effects	Direct	0.89 (0.01)		
	Total indirect	—		
	Total	0.89 (0.01)		
Dung beetle abundance	Direct	0.94 (0.11)	−0.58 (0.08)	
	Total indirect	−0.51 (0.08)		
	Total	0.43 (0.04)		
Dung beetle diversity	Direct	−0.27 (0.22)^NS^	0.24 (0.20)^NS^	0.99 (0.10)
	Total indirect	0.64 (0.26)	−0.58 (0.14)	
	Total	0.37 (0.03)	−0.33 (0.06)	0.99 (0.10)

Abbreviation: NS, not significant.

The direct effects of the forest area and mammal abundance on the diversity of dung beetles was negative, but not statistically significant (ß = −0.27, SE = 0.22, *p* = .23; Table 4; Figure 5), in contrast to the indirect effects which were positive and statistically significant (ß = 0.64, SE = 0.26, *p* = .01). Thus, the total effects were positive and statistically significant (ß = 0.37, SE = 0.03, *p* = .00; Table 4).

The direct relationship between edge effects and the diversity of dung beetles was positive but not statistically significant (ß = 0.24, SE = 0.20, *p* = .22; Figure 5; Table 4), in contrast to the indirect effects which were negative and statistically significant (ß = −0.58, SE = 0.14, *p* = .00). Therefore, the total edge effects on dung beetle diversity were negative and statistically significant (ß = −0.33, SE = 0.06, *p* = .00). Ultimately, the direct effect of the abundance of the dung beetles on their diversity was strong and positive (ß = 0.99, SE = 0.10, *p* = .00), and the model explained most of the variance in the diversity of the beetles (*R* ^2^ = .89).

## 4 DISCUSSION

Our finding that most of the dung beetle species were small (Figure 4, Figure A5) was unexpected for a disturbed forest. Sampling in disturbed forests typically finds inordinate representations of large‐bodied beetles (Nichols et al., 2013), possibly due to two related mechanisms. The bait in pitfall traps constitutes extra, high prized, food resources for all beetles in disturbed habitats (Horgan & Fuentes, 2005). Larger beetles are, however, able to detect and access the bait from much longer distances and are often overrepresented in samples. We, therefore, interpreted the finding that large beetles were rare in these forests to suggest that there is a general scarcity of dung, particularly large piles, which large beetles need to thrive. As a rule, abundant dung is required by dung beetles to metamorphose into large adults (Emlen, 1994; Emlen et al., 2007; Hunt & Simmons, 2000; Iguchi, 1998; Karino et al., 2004; Shafiei et al., 2001). Furthermore, the finding of relatively few large beetles suggests that the two endemic monkeys—the colobus and mangabeys—were probably the primary source of dung resources in the forests. After all, these monkeys are of similarly modest size (body mass, 5–12 kg), and must produce small volumes, and piles, of dung because the body size of a mammal is associated with the amount of dung it produces (Blueweiss et al., 1978).

As we postulated, forest area and mammal abundance were associated positively with the abundance of dung beetles (Figures 1 and 5; Table 4). This is as it should be because dung beetles use the dung from mammals as the food resource of choice and to provision their larvae (Halffter & Edmonds, 1982). This finding is consistent also with the individuals‐area effect, which holds that as the area of habitat fragments increases, then the abundance of resident animals increases (Bender et al., 1998; Connor et al., 2000). In the particular case of the Tana River forests, there is a strong correlation between the number of monkeys and the area of forest fragments (Mbora & Meikle, 2004a; Medley, 1993; Wieczkowski, 2004). This also is consistent with studies from elsewhere which show positive associations between the number of monkeys and the abundance of dung beetles in forest fragments (Culot et al., 2013; Estrada et al., 1999). Accordingly, Edge effects and Dung beetle abundance were negatively associated (Table 4; Figure 5), which is consistent with the individuals‐area relationship too, because as the area of the fragments decreased, their edge effects increased and hence the negative association (Bender et al., 1998; Connor et al., 2000).

Matching with the findings for beetle abundance, forest area, and mammal abundance had positive effects on the diversity of the beetles, and the relationship with edge effects was negative (Table 4; Figure 5). The positive association is probably partly due to the direct effect of the amounts of forest through the species and the individual's area relationships, as expected (Arrhenius, 1921; Bender et al., 1998; Connor et al., 2000; Gleason, 1922). On the one hand, the species‐area effect is a foundational pattern, which could drive direct effects (Arrhenius, 1921). On the other hand, there are indirect effects at play too. Specifically, previous studies in these forests showed that the abundance of trees was associated with the forest area (Wieczkowski, 2004) and that the abundance of monkeys was itself associated with the abundance of the trees (Mbora & McPeek, 2009; Mbora & Meikle, 2004a), because monkeys depend on the trees for food (Medley, 1993; Wieczkowski, 2004). That being so, then the higher abundance of monkeys in larger forests would mean more dung resources, which would promote the species richness of the beetles. This should be the case because resources determine the niche breadths of species at a local scale (MacArthur, 1972). Thus, more species can be found in one area than in another, if more resources are available, if each species overlaps broadly with its neighbors in its resource usage, if each species is more specialized in its resource use, or if the resources dimension is maximally exploited (MacArthur, 1972). Thus, the more the dung resources there were from many more monkeys, as fragments area increased, then the greater the species richness of the beetles.

**FIGURE A9:**
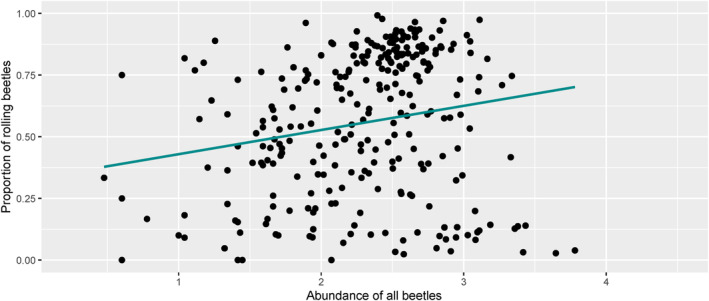
The proportion of rolling beetles increased with the increasing abundance of all beetles (abundance of all beetles is expressed as log base 10 of individuals).

**FIGURE A10:**
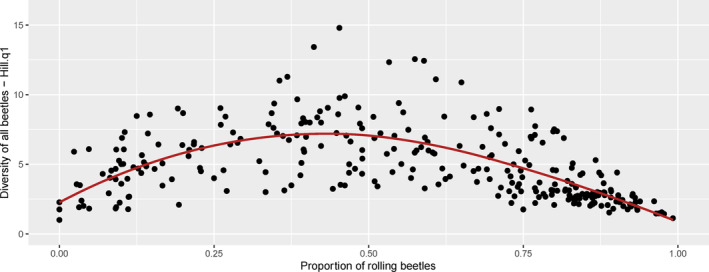
The diversity of dung beetles was maximized at an intermediate level of the proportional representation of rollers (diversity is expressed as effective number of species, Hill.q1).

**FIGURE A11:**
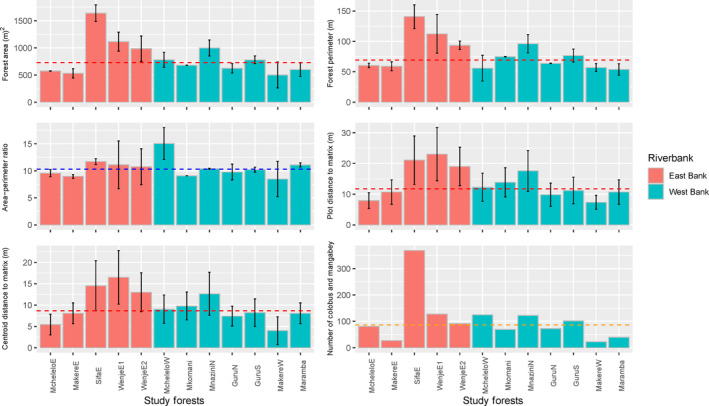
Overview summary of forest attributes, not all of which were used as indicators. All attributes are square root transformed to facilitate comparisons, except for area‐perimeter ratio and number colobus and mangabey. The dashed line represents the median value.

As well, the negative association of the Edge effects and Dung beetle diversity is consistent with the species and the individuals' area relationships because the inverse of the relationships holds just as well. As the area of fragments decreased and so would the species richness and number of individuals, of the beetles. However, as the edge effects increased with the decreasing forest area, other possibly indirect mechanisms, could start to take effect, for example, as proposed by the edge response model (Ries et al., 2017; Ries & Sisk, 2004). For instance, an interaction could occur between edge effects and the beetle nesting strategies because tunneling beetles generally build nests deeper in the soil than rolling beetles do (Halffter & Edmonds, 1982). As a result, larvae of tunnellers would be protected from desiccation, and elevated mortalities, due to edge effects (Murcia, 1995). Indeed, empirical studies have shown that tunneling beetles do better in forests with more edge effects than rolling beetles do (Nichols et al., 2013). This accords with our result that edge effects and dung beetle diversity were inversely related;—perhaps because rolling beetles did not do so well, as edge effects increased. Admittedly, this is not something we tested. However, our data showed that the proportion of rollers increased with the abundance of beetles (Figure A9) and that the overall diversity was maximized at an intermediate proportional mix of rollers (Figure A10). What's more, forests on the west bank which were generally smaller (Figure A11), tended to have more tunnellers than rollers (Figure 4, Figure A5).

Our overarching aim in this study was to use SEM to answer this question: what are the joint effects of the amounts of forest habitat, abundance of mammals, and edge effects on the abundance and diversity of the dung beetles? We surmised that using SEM could provide us with insights on how forest loss and fragmentation jointly influence the abundance and diversity of the beetles, uncover processes that may be operating but are unrecognized, and point to areas needing further studies (Kline, 2016). So then, what did these results tell us about the processes operating in this system? What questions do they raise for further studies?

First, the findings tell us that primates in these forests are crucial to maintaining the abundance and diversity of the dung beetles. This is significant because it is, likely, true of tropical forest fragments elsewhere. Many primates can thrive in forest fragments due to their flexibility of habitat use and monkeys are often the only resident large mammals there. By supporting the dung beetles, the primates are critical to maintaining the forest fragments themselves because the beetles disperse seeds, speed up the cycling of nutrients, and provide many other ecosystem services (Nichols et al., 2008).

Second, the direct effects on the diversity of dung beetles by the factors “Forest area and mammal abundance,” and “Edge effects” were anomalous. The effects of forest area and mammal abundance on the diversity of the beetles were negative, but those of edge effects were positive (Table 4; Figure 5). Both outcomes were not statistically significant, but they merit some conjecture as biologically meaningful because they were contrary to our expectations, and findings from elsewhere (Feer & Hingrat, 2005; Klein, 1989). Consistent with the findings for abundance, these effects imply that as the forest area and mammal abundance decreased—and edge effects increased—and so did the abundance of dung beetles, but that somehow the diversity of the beetles increased. Now how could that occur? As it happened, the smaller forests on the west bank tended to have fewer monkeys but more edge effects (Figure A11). As such, it is possible that the opposite effects were due to a wider variety of dung types in these smaller forests. For example: if wild mammals usually found in the matrix and/or livestock use these forests more regularly. Indeed humans can be a factor too because, in this area, people do use forest edges as a toilet (personal observation, by DNMM).

There is another way by which the factor “Forest area and mammal abundance” could have a negative effect on the diversity, but “Edge effects” have a positive outcome. Specifically, there was an apparent positive association between the proportional the representation of rolling beetles, increasing forest area and abundance of monkeys (Mbora et al. unpublished data; Figure A9). This is consistent, to some extent, with the finding of a positive direct effect of edge effects on diversity, but a negative association of edge effects with abundance (Table 4; Figure 5). Obviously, the increasing proportional representation of one of two main behavioral types necessarily corresponds with reduced diversity overall (Figure A10). Given this finding, there is one question that begs to be answered: in what ways do the functional traits of the beetles influence how they respond to the habitat attributes? While this was not one of the questions we set out to answer, we can conjecture and propose an avenue for future studies.

The use of functional traits to characterize communities in ecology is a growing and promising approach (Brousseau et al., 2018). Functional traits are features of an organism that may influence its performance and/or the ecological processes the organism undertakes (e.g., behavior or nesting strategies for these beetles). The approach makes it possible to consider, at once, the capacity of an organism to survive and reproduce in a habitat, the outcomes of how it interacts with others, and the effects of changes in the habitat. The approach can allow to be discovered the factors that drive the species composition in the first place. Thus, one possible avenue to follow up on our study is to analyze the ways in which forest area, abundance of mammals, and edge effects influence the functional composition of this assemblage of beetles.

Third, our model explained a significant 26% of the variance in the abundance of the beetles, which is considerable for a natural system. However, this implies also that most of the beetle abundance was due to other factors. Now, what factors could those be? We propose that the soil properties are one set of factors that could greatly influence the abundance of the beetles. The dung beetles are soil fauna because the larval and pupal phases are confined entirely within the soil, and constitute more than 50% of the lifespan of the beetles (Halffter & Edmonds, 1982). So, to the extent that soil properties influence the viability of larvae and pupae, then they must influence the abundance of the beetles overall. Therefore, we recommend that future studies test rigorously the ways in which habitat changes influence soil properties, and their effects on the abundance and diversity of the dung beetles.

In conclusion, and in answer to our research question, we found that the amounts of forest habitat, and its fragmented state, influenced the abundance and diversity of the beetles in both direct and indirect ways. Forest area and number of mammals positively influenced the abundance of the beetles, which was the main driver of the diversity. In contrast, edge effects diminished the abundance of the beetles and thereby depressed the diversity.

## AUTHOR CONTRIBUTIONS

**David Nyaga Mugo Mbora:** Conceptualization (lead); data curation (lead); formal analysis (lead); funding acquisition (lead); investigation (lead); methodology (lead); project administration (lead); resources (lead); supervision (lead); validation (lead); visualization (lead); writing – original draft (lead); writing – review and editing (lead). **Morris Nzioka Mutua:** Data curation (supporting); methodology (supporting); validation (supporting).

## FUNDING INFORMATION

This research was made possible by support from Whittier College.

## CONFLICT OF INTEREST STATEMENT

The authors declare that they have no conflict of interest.

### OPEN RESEARCH BADGES

This article has earned an Open Data badge for making publicly available the digitally‐shareable data necessary to reproduce the reported results. The data is available at [https://datadryad.org/stash/share/MROF5BuT74PlfvaMAgEn5cWNAxM7TqS8Kj5prhYVhzk].

## Figures and Tables

**FIGURE A1a:**
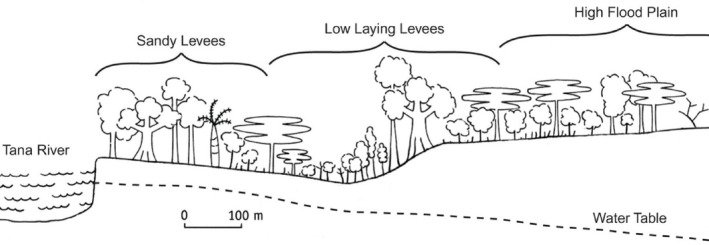
The variation in forest structure along the main environmental gradient, the increasing depth of the water table with distance from the river channel.

**FIGURE A1b:**
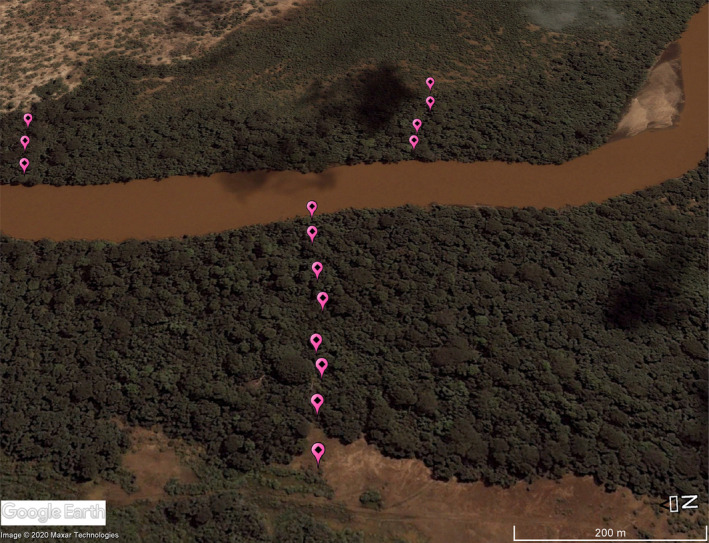
A bird's‐eye view of the orientation of three illustrative transects, across two forests on opposite sides of the river. Pushpins indicate sampling stations.

**FIGURE A2a:**
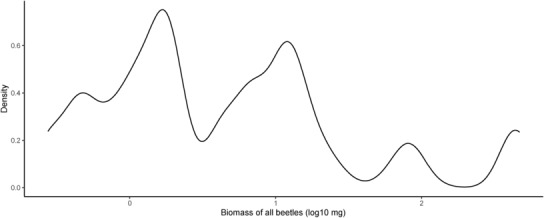
Probability density function of all beetles in the data set, showing the three peaks in the mass of species.

**FIGURE A2b:**
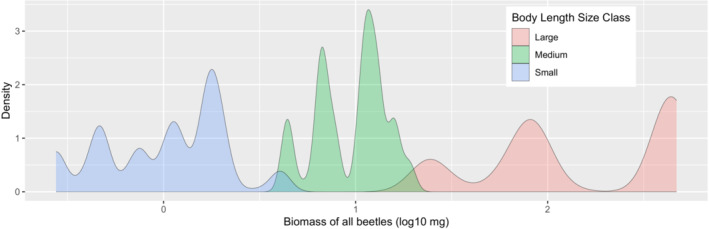
Probability density function with a legend as the back‐transformed body length categories of the beetle body mass. Note that the y axis is a probability density function, that is, the probability of the values in a span of the x axis, and thus values exceed 1.0 where the span of x axis is relatively small.

**FIGURE A3a:**
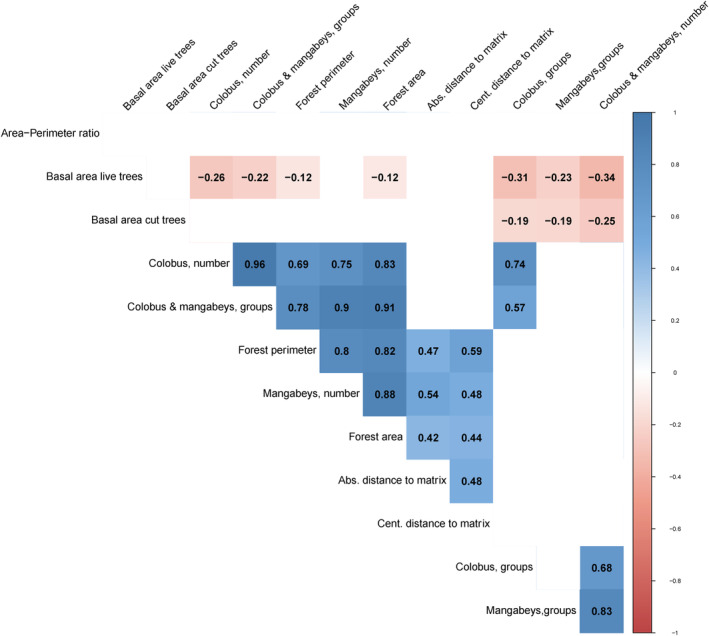
Correlogram of potential indicators of the exogenous factors of the SEM (forests, trees, and monkeys). Numbers indicate correlation coefficient.

**FIGURE A3b:**
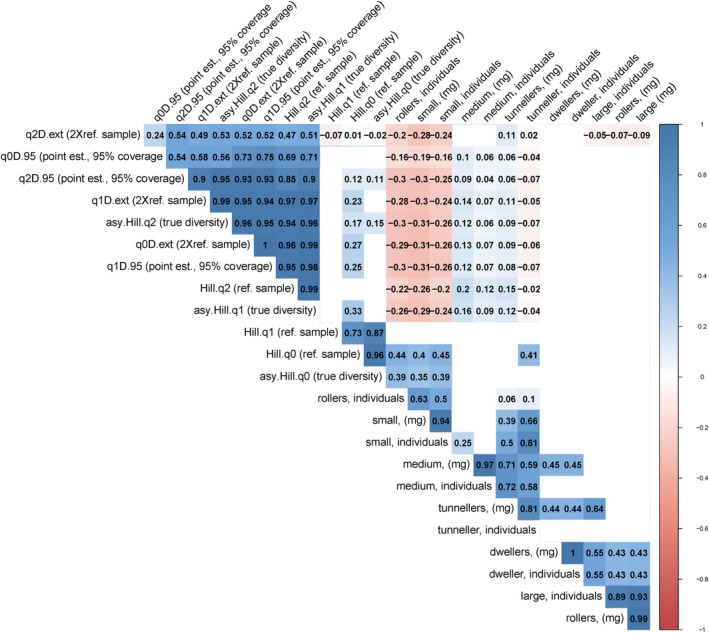
Correlogram of potential indicators of the endogenous factors of the SEM (beetle abundance and diversity). Numbers indicate correlation coefficient.

**FIGURE A4a:**
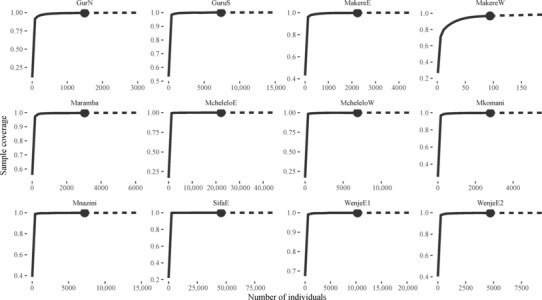
Coverage, which measured sampling completeness, in the 12 study forests.

**FIGURE A4b:**
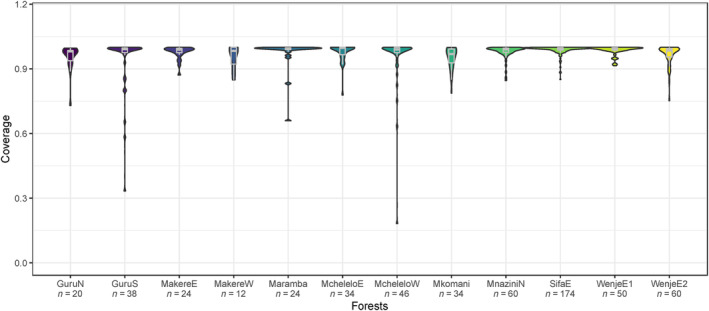
Violin boxplots showing the high sampling completeness (Y axis is % sample coverage) across the 12 study forests. Sample sizes, plots, on the x axis are double the reference sample (details in the text).

## Data Availability

All relevant data produced from this study are provided in this manuscript and its appendices. If any additional data are required, they will be made available at the DRYAD repository: https://doi.org/10.5061/dryad.9cnp5hqkt.

## Footnotes

^1^
This was true only for species represented by at least five individuals.  For species with fewer than five individuals we averaged the length of all individuals.

## APPENDIX A

**TABLE A1:** Summary of attributes of the study forests and dung beetles in the forests.

Attributes	GuruN	GuruS	MakereE	MakereW	Maramba	McheleloE	McheleloW	Mkomani	MnaziniN	SifaE	WenjeE1	WenjeE2
Lat (DMS)	1°51′17″	1°52′20″	1°51′06″	1°50′56″	1°57′02″	1°53′03″	1°53′06″	1°56′43″	1°57′38″	1°53′44″	1°47′14″	1°48′56″
Long (DMS)	40°07′03″	40°08′25″	40°07′02″	40°06′43″	40°08′02″	40°08′31″	40°08′19″	40°08′17″	40°08′10″	40°08′53″	40°07′02″	40°07′12″
Area (m^2^)*	5.58 (0.122)	5.78 (0.08)	5.44 (0.14)	5.31 (0.45)	5.54 (0.18)	5.52 (0.00)	5.77 (0.15)	5.67 (0.00)	5.99 (0.13)	6.42 (0.08)	6.08 (0.14)	5.96 (0.21)
Perimeter (m)*	3.61 (0.00)	3.76 (0.12)	3.54 (0.11)	3.51 (0.10)	3.45 (0.15)	3.56 (0.05)	3.43 (0.33)	3.75 (0.00)	3.95 (0.14)	4.29 (0.12)	4.07 (0.24)	3.94 (0.06)
Area – perimeter ratio*	1.97 (0.13)	2.02 (0.04)	1.91 (0.03)	1.80 (0.035)	2.09 (0.03)	1.96 (0.06)	2.34 (0.18)	1.92 (0.00)	2.03 (0.00)	2.14 (0.04)	2.02 (0.39)	2.02 (0.28)
Plot distance to matrix (m)*	110 (76.56)	143.42 (98.19)	129.17 (83.14)	58.33 (34.16)	129.17 (83.14)	69.12 (42.87)	170.65 (108.62)	213.24 (124.41)	351.67 (217.05)	506.034 (325.31)	601 (366.32)	399.17 (221.13)
Centroid distance to matrix (m)*	60 (35.75)	77.63 (47.79)	70.83 (38.19)	25 (22.36)	70.83 (38.19)	35.29 (21.76)	92.39 (52.46)	105.88 (60.94)	185 (108.20)	245.69 (167.02)	311 (182.87)	189.17 (112.13)
Number of colobus & mangabey	73	102	27	23	40	81	125	70	123	369	128	92
Number of plots	10	19	12	6	12	17	23	17	30	87	25	30
Sum of all beetles	1493	7386	2237	94	3043	22,197	6880	2788	7200	45,642	10,294	4701
Basal area (cm^2^)*	2.99 (1.68)	4.38 (1.97)	3.71 (1.75)	3.41 (2.64)	3.51 (1.66)	2.54 (1.79)	4.04 (1.69)	3.63 (1.02)	3.84 (1.10)	3.72 (1.02)	4.32 (0.95)	5.10 (0.98)
Cut stems (cm^2^)*	0.41 (0.88)	1.55 (1.40)	2.36 (1.23)	2.52 (1.95)	2.27 (1.10)	0.91 (1.35)	0.093 (0.45)	2.61 (0.85)	2.67 (0.79)	1.61 (1.40)	3.15 (0.87)	1.97 (1.49)

*Note*: Forests area and perimeter are expressed as Log_10_ and values tagged with an asterix * are mean (SD).

**TABLE A2:** List of species sampled.

Species name	Mean body length (mm)	Total individuals
1. Sisyphus seminulum (Gerstaecker, 1871)	4.60	42,765
2. Onthophagus variegatus (Fabricius, 1798)	4.11	15,076
3. Onthophagus simplex (Raffray, 1877)	3.11	12,007
4. Onthophagus vinctus (Erichson, 1843)	6.12	6,022
5. Onthophagus raffrayi (Harold, 1886)	4.80	4,723
6. Onthophagus sp. 23	3.39	4,392
7. Caccobius convexifrons (Raffray, 1877)	2.65	3,955
8. Anachalcos convexus (Boheman, 1857)	24.50	3,796
9. Sisyphus sp. 2	6.90	3,464
10. Sisyphus (Neosisyphus) barbarossa (Wiedemann, 1823)	8.14	2,303
11. Sisyphus sp. 5	9.00	2,150
12. Onthophagus omostigma (d'Orbigny, 1902)	3.60	1,965
13. Onthophagus sp. 24	8.49	1,471
14. Proagoderus nigricornis (Fairmaire, 1887)	14.79	1,248
15. Sisyphus rugosus (Gory, 1833)	8.24	1,196
16. Onthophagus metalliger (D'Orbigny, 1913)	7.98	1,129
17. Sisyphus goryi (Harold, 1859)	7.26	626
18. Sisyphus sp. 7	3.93	591
19. Onthophagus tricanniger (d'Orbigny, 1902)	6.92	481
20. Oniticellus spinipes (Roth, 1851)	9.52	472
21. Proagoderus dives (Harold, 1877)	10.21	427
22. Onthophagus multicornis (Orbigny, 1908)	13.40	323
23. Drepanocerus laticolis (Fahraeus, 1857)	3.99	311
24. Sisyphus costatus (Thunberg, 1818)	6.00	256
25. Onthophagus flavibasis (D'Orbigny, 1904)	5.06	253
26. Onthophagus quadrimaculatus (Raffray, 1877)	3.00	230
27. Onthophagus sp. 25	4.00	215
28. Onthophagus viridicatus (Orbigny, 1902)	7.03	188
29. Milichus apicalis (Fåhraeus, 1857)	5.89	187
30. Onthophagus sp. 11	3.00	174
31. Onthophagus sp. 21	2.89	172
32. Proagoderus sexcornutus (d'Orbigny, 1902)	13.74	163
33. Drepanocerus abyssinicus (Rothschild, 1851)	3.49	135
34. Catharsius tricornutus (Degeer, 1778)	15.27	132
35. Onthophagus dohertyi (D'Orbigny, 1902)	10.24	110
36. Onthophagus sp. 19	4.37	101
37. Onthophagus comatulus (D'Orbigny, 1905)	7.00	96
38. Onthophagus sp. 13	7.05	92
39. Caccobius sp. 1	3.13	83
40. Onthophagus sugillatus (Klug, 1855)	6.00	67
41. Onthophagus sp. 22	6.50	42
42. Onthophagus sp. 9	8.00	41
43. Onthophagus teitanicus (d'Orbigny, 1902)	7.34	33
44. Proagoderus extensus (Harold, 1878)	11.37	30
45. Drepanocerus kirbyi (Kirby, 1828)	3.97	27
46. Onthophagus sp. 12	5.50	22
47. Onitis fulgiolus (Klug, 1855)	16.75	20
48. Onthophagus pullus (Roth, 1851)	4.35	18
49. Onthophagus rufonotatus (d'Orbigny, 1902)	8.50	17
50. Onthophagus birugatus (D'Orbigny, 1902)	7.01	14
51. Onthophagus polystigma (D'Orbigny, 1902)	3.60	14
52. Proagoderus versus (d'Orbigny, 1913)	9.20	12
53. Onthophagus laceratus (Peringuey, 1901)	8.13	10
54. Onthophagus sp. 10	8.00	9
55. Onthophagus atrofasciatus (d'Orbigny, 1905)	8.00	7
56. Onthophagus sp. 3	5.50	7
57. Onthophagus sp. 7	8.00	7
58. Gymnopleurus virens (Erichson, 1843)	9.00	6
59. Onthophagus sp. 20	4.37	6
60. Catharsius stuhlmanni (Kolbe, 1893)	25.00	5
61. Sisyphus sp. 6	7.80	5
62. Euoniticellus inaequalis (Reiche, 1847)	8.00	4
63. Milichus sp. 1	9.00	4
64. Onthophagus fuscidorsis (D Orbigny, 1902)	7.80	4
65. Onthophagus sp. 15	5.00	4
66. Onthophagus sp. 2	6.50	4
67. Sisyphus sp. 1	4.50	4
68. Onthophagus sp. 1	8.00	3
69. Onthophagus sp. 18	5.50	3
70. Onthophagus sp. 6	7.50	3
71. Onthophagus sp. 8	7.80	3
72. Sisyphus sp. 3	2.91	3
73. Onthophagus sp. 16	6.00	2
74. Onthophagus sp. 5	8.00	2
75. Digitonthophagus gazella (Fabricius, 1787)	9.50	1
76. Gymnopleurus krugeri (Kolbe, 1895)	11.50	1
77. Gymnopleurus sericiofrons (Fairmaire, 1887)	10.50	1
78. Oniticellus planatus (Castelnau, 1840)	9.50	1
79. Onthophagus picatus (D'Orbigny, 1902)	8.00	1
80. Onthophagus rubricatus (D'Orbigny, 1902)	8.00	1
81. Onthophagus rufobasalis (Fairmaire, 1887)	8.00	1
82. Onthophagus semiasper (D'Orbigny, 1902)	8.00	1
83. Onthophagus sp. 14	8.00	1
84. Onthophagus sp. 17	7.80	1
85. Onthophagus sp. 4	8.00	1
86. Proagoderus sp. 1	7.00	1
87. Sisyphus sp. 4	4.85	1

**TABLE A3:** Global fit statistics of a two‐step testing of the five‐factor structural regression model of the determinants of dung beetle abundance and diversity.

Model	χ^2^	df	*p*	χ^2^_D_	df_D_	*p*	RMSEA (90% CI)	CFI	SRMR
Measurement model									
One factor—CFA	1490.76	65	.00				0.276 (0.264–0.288)	0.512	0.200
Five factors—CFA	497.59	56	.00	993.17	9	.000	0.165 (0.152–0.165)	0.949	0.089
Five‐factor CFA (ids = ~east.west)	67.25	56	.144 (.472*)	430.34	0	.000	0.026 (0.035–0.016)	0.982	0.083
Structural regression model									
Nested (ids = ~forest)	92.24	56	.144 (.206*)				0.047 (0.040–0.055)	0.843	0.083
Nested (ids = ~east.west)	67.25	56	.002 (.472*)				0.026 (0.035–0.016)	0.982	0.083

* The *F*‐reference distribution for *p*‐value; *ids* specifies the survey design.

**TABLE A4:** Robust Maximum likelihood estimates of pattern coefficients and residuals of a five‐factor measurement model of the determinants of dung beetle abundance and diversity.

	Pattern coefficients				Error variances			
	Unstandardized		Standardized		Unstandardized		Standardized	
Factors and their indicators	Estimate	SE	Estimate	SE	Estimate	SE	Estimate	SE
Dung beetle abundance (mass, mg.)								
Large body size	1.000	—	0.699	0.033	1.347	0.122	0.512	0.046
Medium body size	0.457	0.036	0.806	0.024	0.145	0.145	0.351	0.039
Tunneling behavior	0.581	0.043	0.844	0.021	0.175	0.019	0.287	0.035
B Dung beetle diversity								
Hill.q0	1.000	—	0.978	0.013	1.185	0.693	0.043	0.026
Hill.q1	0.018	0.003	0.399	0.051	0.045	0.004	0.840	0.041
Asymptotic Hill.q0	0.040	0.002	0.806	0.023	0.022	0.002	0.350	0.038
C Mammals: abundance & species richness								
Colobus & mangabey—individuals	1.000	—	0.981	0.009	0.004	0.002	0.037	0.017
Colobus & mangabey—groups	0.210	0.021	0.516	0.044	0.014	0.001	0.734	0.046
Mangabeys—individuals	0.926	0.040	0.841	0.019	0.040	0.004	0.293	0.032
D Forest: spatial attributes								
Forest area (m^2^)	1.000	—	1.000	0.000	0.000	—	0.000	0.000
E Edge effects: microclimate & boundary								
Absolute distance to matrix	1.000	—	0.501	0.046	0.169	0.014	0.749	0.046
Centroid distance to matrix	17.948	2.404	0.592	0.041	33.830	2.938	0.649	0.048
Forest perimeter	1.379	0.152	0.957	0.019	0.010	0.004	0.084	0.036

**TABLE A5:** Robust maximum likelihood estimates of factor variances and covariances for a five‐factor measurement model of the determinants of dung beetle abundance and diversity.

Parameter	Unstandardized		Standardized	
	Estimate	Standard error	Estimate	Standard error
Dung beetle abundance (DBA)	1.284	0.195	1.000	0.000
Dung beetle diversity (DBD)	26.148	2.377	1.000	0.000
Mammals: abundance & species richness (MASR)	0.112	0.010	1.000	0.000
Forest: spatial attributes (FSA)	0.138	0.012	1.000	0.000
Edge Effects: microclimate & boundary (EE)	0.057	0.013	1.000	0.000
DBA ~~ DBD	5.436	0.587	0.938	0.019
DBA ~~ MASR	0.183	0.029	0.483	0.052
DBA ~~ FSA	0.132	0.029	0.312	0.059
DBA ~~ EE	0.069	0.020	0.255	0.063
DBD ~~ MASR	0.693	0.112	0.405	0.052
DBD ~~ FSA	0.537	0.119	0.282	0.056
DBD ~~ EE	0.311	0.085	0.256	0.059
FSA ~~ MASR	0.112	0.010	0.896	0.014
EE ~~ MASR	0.064	0.009	0.891	0.021
FSA ~~ EE	0.079	0.011	0.801	0.029

**TABLE A6:** Matrix of correlation residuals, for the selected four‐factor model of the determinants of dung beetle abundance and diversity.

Factors and their indicators	Large body size	Tunneling behavior	Medium body size	Hill.q0	Asymptotic Hill.q0	Hill.q1	Absolute distance to matrix	Centroid distance to matrix	Forest perimeter	Forest area	Colobus & Mangabey, individuals	Mangabey, individuals
Dung beetle abundance												
Large body size	0.00											
Tunneling behavior	0.13	0.00										
Medium body size	−0.12	−0.02	0.00									
B Dung beetle diversity												
Hill.q0	−0.03	−0.01	0.03	0.00								
Asymptotic Hill.q0	−0.01	−0.01	−0.02	0.00	0.00							
Hill.q1	0.04	0.04	0.05	−0.01	0.02	0.00						
C Edge effects												
Absolute distance to matrix	0.25	0.19	0.08	0.08	0.14	−0.09	0.00					
Centroid distance to matrix	−0.15	−0.22	−0.03	−0.09	−0.03	−0.06	−0.07	0.00				
Forest perimeter	0.15	−0.05	−0.02	0.00	0.02	−0.07	−0.01	0.01	0.00			
D Area & resources												
Forest area	0.09	−0.15	−0.07	−0.07	−0.03	−0.09	0.10	−0.01	0.06	0.00		
Colobus & Mangabey, individuals	0.23	−0.01	0.02	0.04	0.00	0.02	0.02	−0.07	−0.04	0.00	0.00	
Mangabey, individuals	0.14	0.01	0.12	0.07	0.10	0.00	0.09	0.01	−0.07	−0.03	0.04	0.00

*Note*: Highlighted values are >|0.1| (Kline, 2016). Indicator names are as described in full in Table 2. Also, see Figure A8.
